# Orbital inflammation following COVID-19 vaccination: A case series and literature review

**DOI:** 10.1007/s10792-023-02747-6

**Published:** 2023-05-17

**Authors:** Terence Ang, Jessica Y. Tong, Sandy Patel, Jwu Jin Khong, Dinesh Selva

**Affiliations:** 1 Department of Ophthalmology and Visual Sciences, The University of Adelaide, Adelaide, South Australia Australia; 2 Department of Ophthalmology, Royal Adelaide Hospital, Adelaide, South Australia Australia; 3 Department of Medical Imaging, Royal Adelaide Hospital, Adelaide, South Australia Australia; 4 Orbital, Plastics and Lacrimal Unit, Royal Victorian Eye and Ear Hospital, Melbourne, VIC Australia

**Keywords:** Orbit, Orbital inflammation, Orbital myositis, Dacryoadenitis, COVID-19, Vaccine

## Abstract

**Purpose:**

The purpose of the study was to report three cases of orbital inflammation following administration of the COVID-19 vaccination, manifesting as Tolosa–Hunt syndrome (THS) and orbital myositis.

**Method:**

A retrospective case series and literature review of patients who developed orbital inflammation following a COVID-19 vaccination.

**Results:**

One patient presented with Tolosa–Hunt syndrome (THS) 14 days following her third (booster) COVID-19 vaccination, one patient developed orbital myositis 10 days following his first COVID-19 vaccination and one patient developed recurrent orbital myositis 1 and 7 days following her second and fourth COVID-19 vaccination. All patients received the Comirnaty vaccine (Pfizer-BioNTech). A thorough systemic autoimmune disease workup in both patients was unremarkable. Two patients had a prior history of orbital inflammation, with previous involvement of other different orbital structures. Characteristic MRI features for each pathology were observed, supporting the clinical presentation of THS and orbital myositis. There was complete resolution of THS following corticosteroids, with no recurrence at 2 months. Meanwhile, one case of orbital myositis self-resolved at 2 months without use of systemic corticosteroids, while the other patient with orbital myositis required treatment with intra-orbital steroid injections and oral corticosteroids.

**Conclusion:**

Orbital inflammation has been recognised as a rare adverse effect following COVID-19 vaccination. We present a case series of THS and orbital myositis as varied presentations of this entity.

## Introduction

Recent literature has reported various ophthalmic adverse effects associated with the administration of COVID-19 vaccinations (Pfizer-BioNTech, Moderna, AstraZeneca, SinoPharm and SinoVac) [1–3]. To date, there have been 11 reported cases of COVID-19 vaccine-associated orbital inflammation, including six cases of isolated myositis, two cases each of Tolosa–Hunt syndrome (THS) and diffuse orbital inflammation and one case of isolated dacryoadenitis [4–11].

We present a case series of COVID-19 vaccine (Comirnaty, Pfizer-BioNTech)-associated orbital inflammation, including THS and orbital myositis. We also outline the clinico-radiological features of this rare adverse effect of the COVID-19 vaccine.

## Method

A retrospective case series of patients who developed orbital inflammation within 21 days following administration of the COVID-19 vaccination. Patients with clinico-radiological evidence of inflammation of any orbital structure (e.g. orbital fat, extraocular muscles, lacrimal gland, etc.) were included. A comprehensive literature search was conducted on PubMed for cases of COVID-19 vaccination-associated orbital inflammation, and relevant articles from reference lists were also included. This case series adheres to the tenets outlined in the Declaration of Helsinki. Written informed consent was obtained from the patients for publication and use of clinical photographs.

## Results

### Case 1

A 58-year-old woman presented with a 3-week history of the left periorbital pain, ptosis and painful ophthalmoplegia. Her symptoms began with the left nasal pain that worsened with Valsalva manoeuvres and was unresponsive to oral Amoxicillin. One week later, she developed increasing left periorbital and retrobulbar pain. She subsequently developed a cranial nerve III palsy with painful left eye ophthalmoplegia, diplopia and ptosis.

Fourteen days prior to the symptom onset, she had received her third COVID-19 vaccination (Comirnaty, Pfizer-BioNTech). There were no adverse effects from her two previous COVID-19 vaccinations (Comirnaty, Pfizer-BioNTech) 4 and 5 months prior. Six years prior, she had a single episode of biopsy-proven bilateral idiopathic dacryoadenitis that completely resolved with corticosteroids. She was on no regular medications and had not received any other recent vaccinations.

**Fig. 1:**
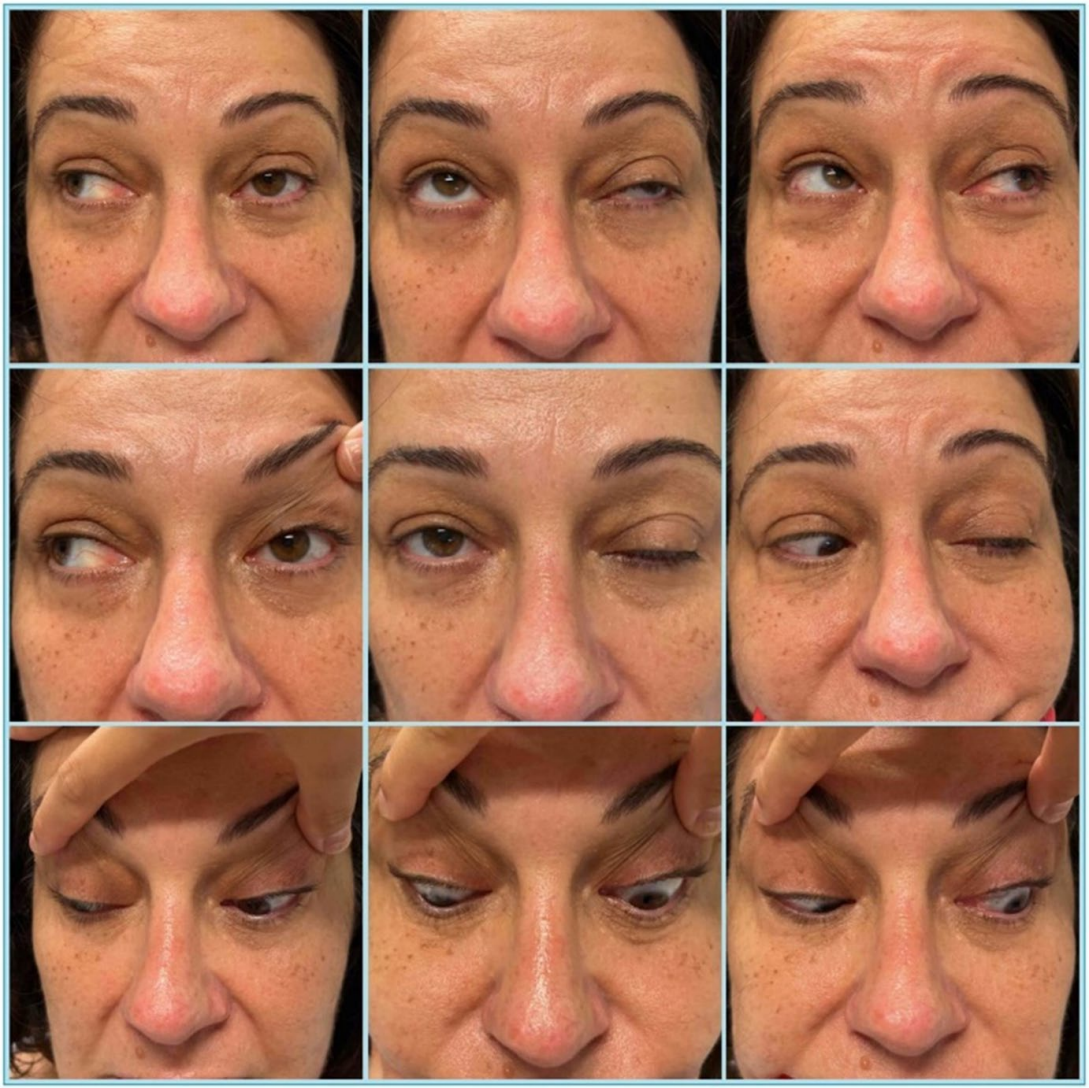
Clinical photographs of Case 1 at initial presentation demonstrating left cranial nerve III palsy. There is ptosis with restriction in the left eye adduction, elevation and depression

On examination, visual acuity (VA) was 6/4.5 bilaterally, with normal intraocular pressures (IOP). External evaluation revealed left upper lid partial ptosis, but no periocular erythema, oedema or proptosis. Ocular motility examination revealed a cranial nerve III palsy with 15 prism dioptres of the left exotropia and hypotropia (Fig. 1). Optic nerve function was normal. Anterior and posterior segment examination was unremarkable. At subsequent review, she reported new-onset altered sensation in the cranial nerve V_1_ distribution.

**Fig. 2:**
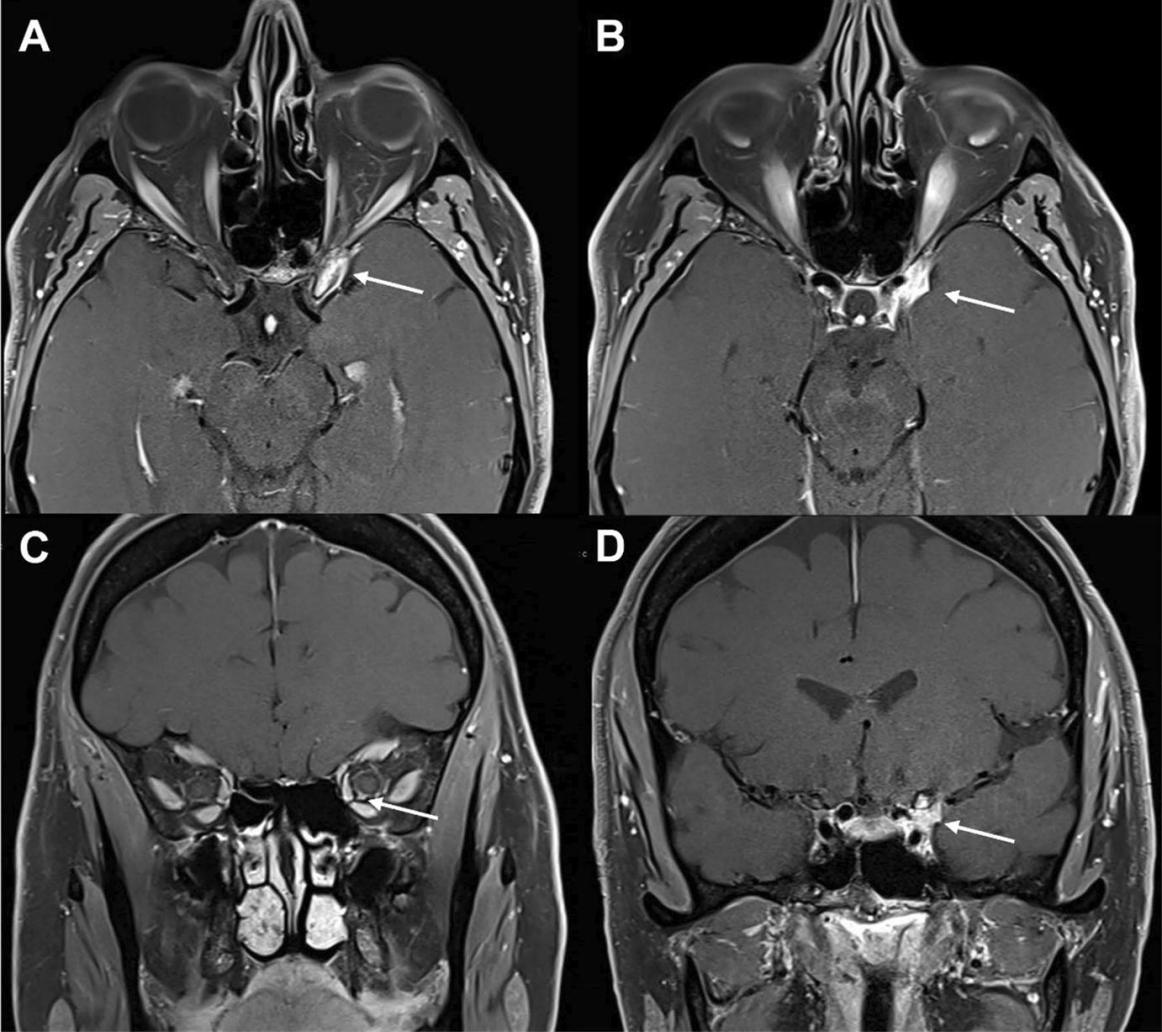
T1-weighted, fat-suppressed post-contrast MRI scans for Case 1, demonstrating the enhancing infiltration of the left cavernous sinus with extension through the superior orbital fissure to orbital apex with apical nerve sheath enhancement (white arrows)

Initial investigations revealed normal inflammatory markers: white cell count (WCC) 8.57 × 10^3^/μL, C-reactive protein (CRP) 1.7 mg/L and erythrocyte sedimentation rate (ESR) 9 mm/hr. Autoimmune serology (ACE, ANA, ANCA and ENA) and IgG4 levels were unremarkable, and there was no atypical expression of cell lymphoid markers. MRI orbital scans revealed enhancement and enlargement of the left cavernous sinus and clinoid, with dural enhancement and extension through the superior orbital fissure to the orbital apex with mild apical nerve sheath enhancement (Fig. 2A–D). The lobulated appearance, lack of hyperostosis of the anterior clinoid and the enhancing clinoid were less consistent with meningioma.

The clinico-radiological features were consistent with THS, likely precipitated as an autoimmune phenomenon in context of her recent COVID-19 vaccination. She was commenced on a 1-week course of 80 mg of oral prednisolone (1 mg/kg), before being tapered by 10 mg weekly until 20 mg. This was followed by a 5 mg weekly taper to 5 mg on alternate days before cessation. There was significant improvement in pain after 4 days, with complete resolution of diplopia and ptosis and no recurrence at final follow-up 2 months later. A neurologist was consulted for confirmation of THS, and it was deemed that following the rapid response to systemic corticosteroids, a lumbar puncture was not indicated unless there was atypical resolution or recurrence.

### Case 2

**Fig. 3:**
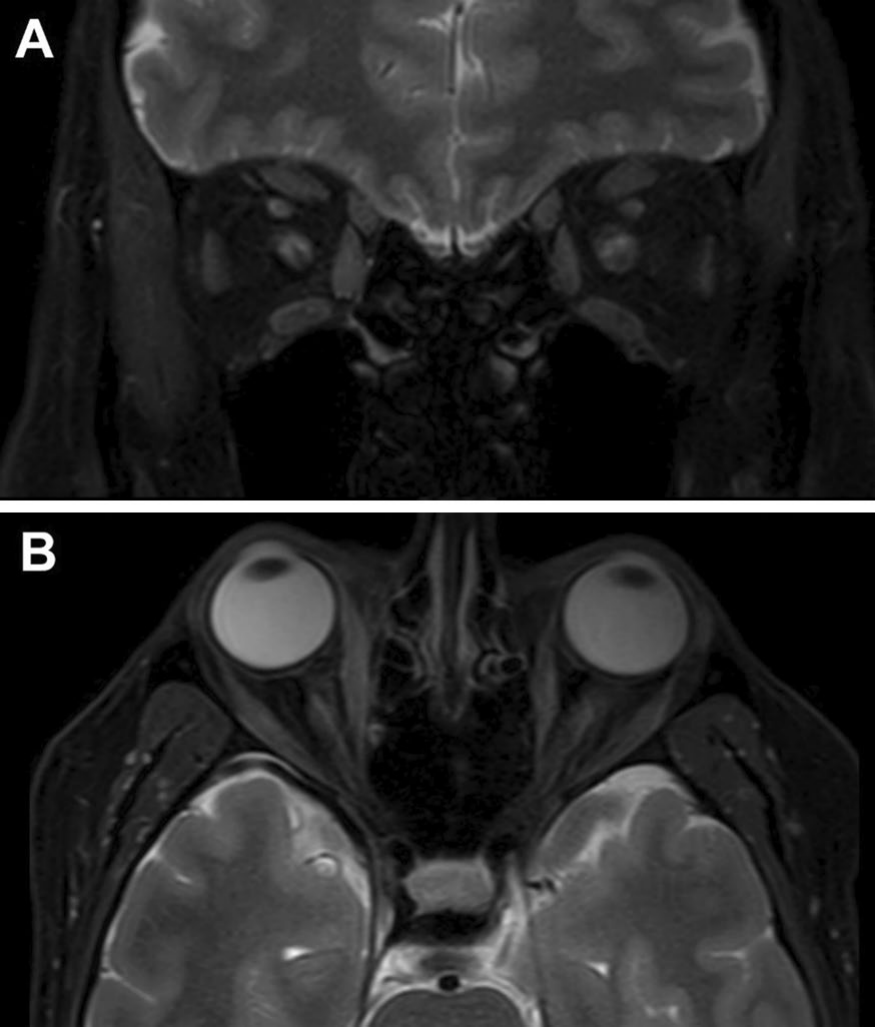
T2-weighted, fat-suppressed MRI scans for Case 2. A (coronal) and B (axial) demonstrate the predominant asymmetric thickening of the right medial rectus with tendon involvement evidenced by T2 hyperintensity, along with bilateral enlargement of the lateral and inferior recti

A 46-year-old male presented with a 2-week history of episodic right periorbital oedema and erythema. Symptoms began 10 days after his first COVID-19 vaccination (Comirnaty, Pfizer-BioNTech). On initial examination, additional to the periorbital oedema and erythema, there was right medial conjunctival and episcleral injection. There was no proptosis, and oculomotility was full. He was diagnosed with episcleritis and commenced on topical dexamethasone, with significant clinical improvement. Past medical history included a right radial keratotomy, right ptosis repair and left idiopathic episcleritis. He was on no regular medications. There was normal thyroid function, including negative thyroid autoantibodies. An autoimmune screen detected ANCA positivity, but absent proteinase-3 and myeloperoxidase. MRI orbital scans revealed predominant asymmetric thickening of the right medial rectus with tendon involvement evidenced by T2 hyperintensity, along with bilateral enlargement of the lateral and inferior recti (Fig. 3). This clinico-radiological presentation was consistent with orbital myositis. Conservative management with selenium supplements was commenced, and a second opinion was sought 2 months after onset. At subsequent review, there was significant clinical improvement, with no signs of active inflammation or recurrence. On examination, the right and left VA (pinhole) was 6/9 and 6/6, respectively, with normal IOP bilaterally. There was full range of extraocular movements, normal optic nerve function and no lid retraction or proptosis. A biopsy and commencement of systemic corticosteroids were not indicated given the self-resolving episode, and the patient remained completely asymptomatic at 4 months.

### Case 3

**Fig. 4:**
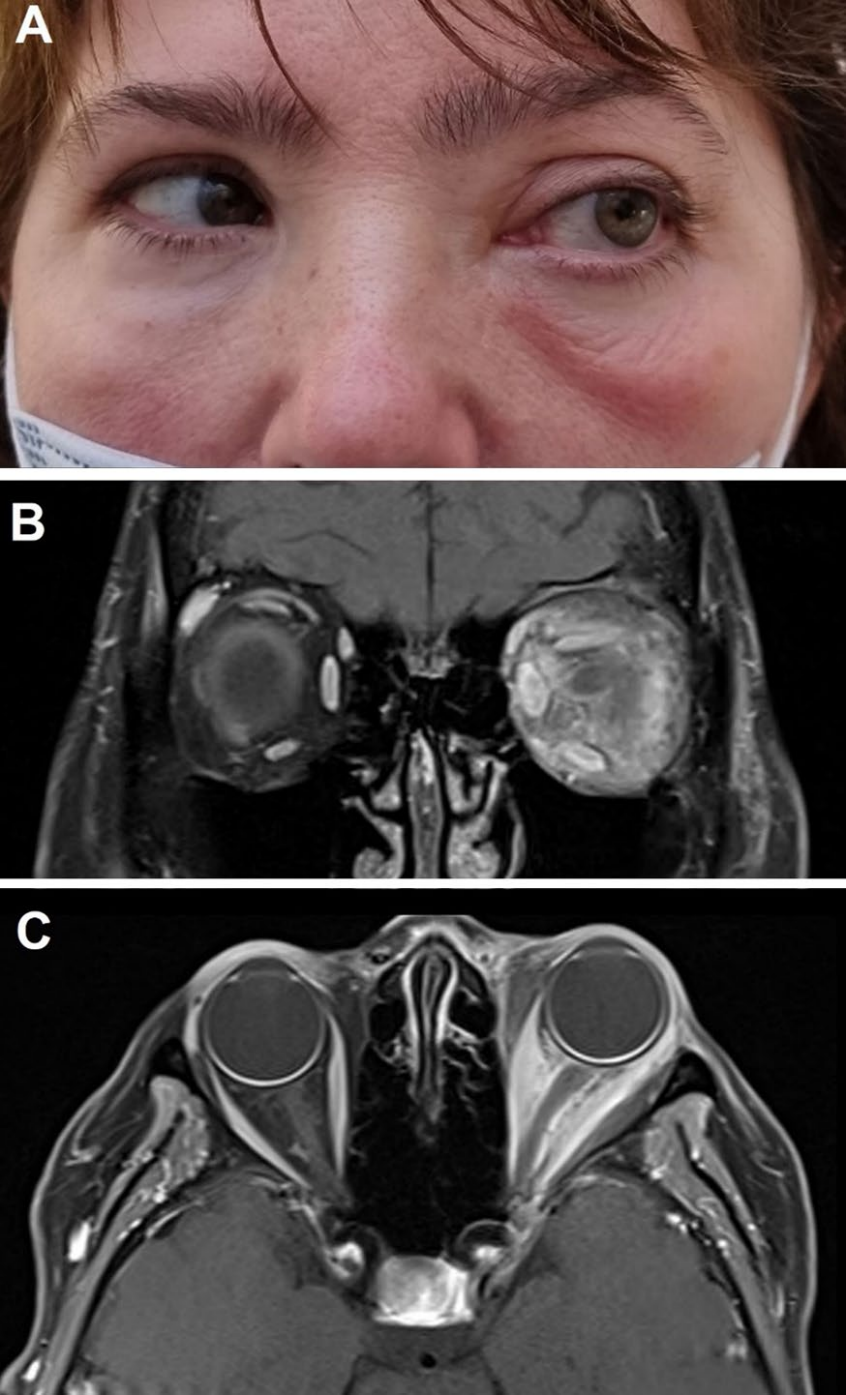
Clinical photographs and MRI orbits of Case 3. A demonstrates the surrounding periorbital oedema and erythema, with proptosis and restricted left eye abduction. B (coronal) and C (axial) demonstrate T1-weighted, fat-suppressed, contrast-enhanced images showing proptosis and diffuse extra- and intra-conal fat enhancement extending close to the orbital apex and superior orbital fissure, with enlargement and peripheral enhancement of all the EOM

A 61-year-old female presented with two recurring episodes of increased left periorbital oedema, discomfort, vertical diplopia and proptosis. The first episode occurred 1 day after her second COVID-19 vaccination (Comirnaty, Pfizer-BioNTech) and was treated with intra-orbital steroid injections. There were no adverse effects from her first COVID-19 vaccination. She experienced no symptoms following her third COVID-19 vaccination, however, was receiving concurrent treatment of methotrexate and tracheal steroid injections for recurrent laryngitis. Due to subsequent mild liver enzyme elevation, methotrexate was ceased for a month. However, during this time, and coinciding with 7 days after her fourth COVID-19 vaccination, she presented with a recurrent episode of left-sided superior and supero-temporal periorbital oedema and erythema, diplopia and proptosis and reduced left eye abduction (Fig. 4A). This was managed by an intra-orbital steroid injection and an oral course of 50-mg prednisolone which was followed by a 5-week taper, before she was recommenced on 10-mg methotrexate weekly.

Relevant past medical history included recurrent episodes of biopsy-confirmed non-specific isolated left dacryoadenitis diagnosed 18 years prior which was completely responsive to oral prednisolone. She also had biopsy-confirmed granulomatous iritis and recurrent laryngitis requiring a biopsy which revealed granulation tissue with multinucleate giant cells, but no definite vasculitis. This medical history along with a previously elevated pANCA level with positive MPO, raised clinical concern for granulomatous polyangiitis (GPA), although no definite diagnosis had been confirmed by rheumatology. Her left dacryoadenitis, periorbital swelling and recurrent stridor appeared to be manifestations of a presumed granulomatous disease process. Remaining autoimmune screen and inflammatory markers were unremarkable.

Initial MRI orbital scans revealed proptosis and extra- and intra-conal fat enhancement extending toward the orbital apex, superior orbital fissure and anterior cavernous sinus, without thrombosis (Fig. 4B and C). There was enlargement and peripheral enhancement of all the extraocular muscles (EOM), including the posterior superior muscle complex and superior oblique, distal inferior oblique and the recti and superior oblique. The left lacrimal gland was poorly defined and atrophic, consistent with prior recurrent dacryoadenitis. At subsequent review, 14 months after her initial episode, she remained stable with no major changes in her localised disease, with further monitoring for systemic vasculitis disease.

## Discussion

COVID-19 vaccine-associated ophthalmic adverse effects have been reported in recent literature. These adverse effects include uveitis, episcleritis and scleritis, optic and cranial neuropathies, intraretinal changes and orbital inflammation including acute-onset dacryoadenitis, myositis and THS [2, 4, 8, 12–18]. Symptom onset ranged from 9 h up to 42 days post-vaccination [1, 4, 6, 7, 19]. To the best of our knowledge, this case series represents the third reported occurrence of THS and two additional reports of orbital myositis following the COVID-19 vaccine (Comirnaty, Pfizer-BioNTech) [4, 6, 8, 19]. Table 1 summarises the cases of this present study and those reported in the literature.

**Table 1:** Summary table of cases of COVID-19 vaccination-associated orbital inflammation. Cases 1–3 represent the present study’s cases.

Case (Reference)	Gender/age	Prior history of orbital inflammation	Vaccination	Dose	Time to onset	Laterality	Clinical features							Radiological features			
							Periorbital erythema/oedema	Conjunctival injection/chemosis	Painful/restricted EOM	Diplopia	Proptosis	Optic neuropathy/reduced VA	Other symptoms	Orbital fat inflammation	EOM enlargement	Lacrimal gland inflammation	Other
1	F/58	+	BNT162b2 mRNA, Pfizer/BioNTech	3	14d	Left	+		+	+				+			Orbital apex extending to SOF and cavernous sinus
2	M/46		BNT162b2 mRNA, Pfizer/BioNTech	1	10d	Right	+	+					Episcleritis		+ (Bilateral)		
3	F/61	+	BNT162b2 mRNA, Pfizer/BioNTech	2 and 4	1d and 4d	Left	+		+	+	+			+	+		
4 [8]	M/68		BNT162b2 mRNA, Pfizer/BioNTech	2	4d	Left	+		+	+	+				+		
5 [8]	F/33	+ *	mRNA-1273, Moderna	2	1d	Left	+	+	+	+	+		Myalgia, headache		+		
6 [8]	M/13	+	BNT162b2 mRNA, Pfizer/BioNTech	1	1d	Left	+	+	+		+			+		+	
7 [6]	F/39	+ ^#^	mRNA-1273, Moderna	1	24 h	Bilateral	+	+	+		+	+			+		
8 [7]	M/14		BNT162b2 mRNA, Pfizer/BioNTech	1	9 h	Right	+	+	+	+	+			+	+	+	
9 [10]	F/40		BNT162b2 mRNA, Pfizer/BioNTech	1	7d	Left	+				+				+	+	
10 [9]	F/65		BNT162b2 mRNA, Pfizer/BioNTech	1 and 2	2d	Bilateral	+	+			+	Right-side		+	+		
11 [4]	M/45		mRNA-1273, Moderna	1	5d	Left			+	+		+	Ptosis, headache				Orbital apex, extending to cavernous sinus
12 [14]	F/76		CoronaVac/Sinovac	2	35d	Right			+	+			Preceding headache, photophobia, phonophobia; ptosis				Cavernous sinus
13 [11]	F/64		BNT162b2 mRNA, Pfizer/BioNTech	2	5d	Right	+	+	+	+	+		Scleritis		+		
14 [11]	F/45		mRNA-1273, Moderna	1	6d	Left			+	+	+		Asthenopia when reading		+		

*Episode of orbital inflammation 1 day following influenza vaccination occurring 1 year prior.

^#^Ten-year history of mild episodic pre-septal periocular swelling lasting 1–2 weeks and occurring approximately once a year, self-resolving and without any residual visual compromise

Tolosa–Hunt syndrome has recently been described following both COVID-19 infections and vaccinations [4, 5]. Chuang et al. first reported THS in a 45-year-old male, 7 days after his first dose of COVID-19 vaccination (mRNA-1273) [4]. Contrast-enhanced MRI orbits revealed bilateral perineural enhancement surrounding the optic nerve sheaths (left greater than right), with poorly-defined enhancement in the left orbital apex extending into the cavernous sinus [4]. Pedro et al. similarly reported occurrence of THS in a 76-year-old female after completing both doses of the CoronaVac/Sinovac COVID-19 vaccination scheme (45 days between the first and second doses). The patient initially experienced multiple episodes of severe headache, phonophobia, photophobia and otalgia, shortly after the first dose and worsening after the second dose. Symptoms of THS, including painful diplopia with incomplete palsy of the right cranial nerves III, IV and VI, occurred 35 days after the second dose [19]. In contrast with the previous two reported cases, onset of THS in our patient only occurred after the third (booster) vaccination with no adverse effects after the initial two doses.

Inflammation following COVID-19 vaccination has been reported to affect various orbital structures, including six cases of isolated myositis, two cases of diffuse orbital inflammation (lacrimal gland, extraocular muscle, and orbital fat involvement) and one case of isolated dacryoadenitis (Table 1) [6–11]. Within the literature and our cases, orbital inflammation is primarily unilateral, predominantly involve the extraocular muscles and orbital fat, while lacrimal gland is less commonly involved. Acute bilateral orbital myositis was reported 24 h after administration of mRNA-1273 (Moderna) vaccine [6]. Additionally, Gencoglu and Mangan described a case of orbital inflammation, affecting the lateral and superior recti, and lacrimal gland, following the first dose of COVID-19 vaccination (Comirnaty, Pfizer-BioNTech) 1 week prior [10]. Orbital myositis may also present with scleritis or episcleritis, as described by Savino et al. and within our case (Case 2) [11]. Reshef et al. described three patients with orbital involvement, ranging from the EOM, lacrimal gland and intraconal fat, all occurring within 1 week of vaccination [8]. Meanwhile, in our case of orbital myositis, onset of symptoms was observed 10 days following the first dose. Grunewald et al. have reported bilateral orbital inflammation involving the EOM and orbital fat occurring approximately 2 days after the first COVID-19 vaccination (Comirnaty, Pfizer-BioNTech), with exacerbation of the right-sided symptoms occurring after the second dose [9]. Similarly, exacerbation of orbital inflammation coincided with administration of the fourth COVID-19 vaccination in Case 3. Finally, Murphy et al. described acute-onset dacryoadenitis occurring 9 h following the first dose of COVID-19 vaccination (Comirnaty, Pfizer-BioNTech) [7].

The duration between COVID-19 vaccination and symptom onset of orbital inflammation is variable. In the two reported cases of THS, ocular symptoms occurred 5 and 35 days after vaccination, respectively [4, 19]. Meanwhile, our patients developed symptoms 1–14 days after their vaccination. Other cases of orbital inflammation have generally had more acute onset, ranging from 9 h to 4 days after vaccination [6–8]. Nevertheless, late-onset adverse effects, up to 42 days following COVID-19 vaccination, with signs of intraocular inflammation, have been reported; however, delayed presentation could not be excluded [3]. In this particular case of THS, we postulate that repeated exposure to an immunogenic stimulus, such as the COVID-19 vaccination, may have precipitated orbital inflammation following the third (booster) dose. However, we cannot comment on the causality of Tolosa–Hunt syndrome in this case, and it remains entirely plausible that it occurred as a vaccination-independent phenomenon, as evident by a number of orbital inflammation cases presenting after the first dose of COVID-19 vaccination.

The majority of patients with uveitis and scleritis occurring after a COVID-19 vaccination had previous episodes of an ocular inflammatory event [13]. This has also been reported in the orbital inflammation literature [20]. Reshef et al. reported two patients with prior episodes of non-specific orbital inflammation. One patient had recurrent myositis triggered by upper respiratory tract infections, while the other had previously experienced orbital inflammation following the influenza vaccine 1 year prior [8]. In our patient with THS, however, a different manifestation of orbital inflammation was observed from the initial episode of bilateral idiopathic dacryoadenitis 6 years prior. Our patient in Case 2 had a previous history of idiopathic episcleritis. Meanwhile, Case 3 had recurrent episodes of steroid-responsive biopsy-confirmed non-specific dacryoadenitis, although there was a suspicion of an underlying granulomatous disease process. Thus, it remains plausible that certain stimuli, such as the COVID-19 vaccine, may incite inflammation in patients with a tendency for both non-specific and specific causes of orbital inflammation. Patients with prior orbital inflammation may have an autoimmune predisposition to certain stimuli such as the COVID-19 vaccination, although a statistically significant correlation has not been observed [21]. Additionally, both the COVID-19 infection and other vaccines for influenza and shingles have also been associated with occurrence of orbital inflammation [22–26]. Manifestations of these *de novo* cases of orbital inflammation included sudden-onset unilateral ptosis and orbital myositis, bilateral orbital inflammation and orbital myositis with posterior scleritis [22–26].

Several hypotheses, primarily revolving around an immunological process, have been proposed regarding the pathogenesis of COVID-19 vaccine-associated orbital inflammation [6, 8]. Our case series, along with eight other reported cases, have been associated with an mRNA-based COVID-19 vaccine. It has been postulated that uncoated mRNA molecules are pro-inflammatory in the extracellular compartment and degradation of the coated mRNA vaccine, may incite inflammation [27]. Other proposed mechanisms implicate the vaccine’s excipient components [6, 28]. We acknowledge that with mass administration of the COVID-19 vaccination programme in Australia, it remains plausible that orbital inflammation may only be coincidentally associated with recent vaccination.

Management of COVID-19 vaccine-associated orbital inflammation included a course of intravenous and/or oral corticosteroid, or oral nonsteroidal anti-inflammatories (NSAIDs) [4, 6–11]. Case 2 had complete resolution with conservative measures alone. Depending on the severity of inflammation and status of optic nerve function, corticosteroid regimens included 1-g intravenous (IV) methylprednisolone for 3 days, followed by an oral corticosteroid taper over 6 weeks, or 60-mg oral prednisone which was tapered over 1 month [4, 6–8]. Case reports have also described the use of prophylactic corticosteroids before subsequent COVID-19 vaccinations, with regimens describing continuing the tapering dose from the initial episode at 20 mg prior to the second vaccination; or initiating a 3-day course of 60-mg oral prednisolone followed by a 10-mg taper every 3 days, with no further episodes of orbital inflammation.

In conclusion, we reported three cases of orbital inflammation occurring 1–14 days following a COVID-19 vaccination (Comirnaty, Pfizer-BioNTech). The clinical manifestations included THS and isolated orbital myositis, and these cases add to a steadily growing body of the literature of COVID-19 vaccine-associated orbital inflammation. The causal association cannot be determined but recognition of this entity is important in addition to consideration of future vaccinations for such patients.
